# Major ambulatory surgery among US adults with inflammatory bowel disease, 2017

**DOI:** 10.1371/journal.pone.0264372

**Published:** 2022-02-24

**Authors:** Fang Xu, Anne G. Wheaton, Yong Liu, Kurt J. Greenlund

**Affiliations:** Division of Population Health, National Center for Chronic Disease Prevention and Health Promotion, Centers for Disease Control and Prevention, Atlanta, GA, United States of America; Auburn University, UNITED STATES

## Abstract

**Background:**

Patients with inflammatory bowel disease (IBD) have higher health services use than those without IBD. We investigated patient and hospital characteristics of major ambulatory surgery encounters for Crohn’s disease (CD) or ulcerative colitis (UC) vs non-IBD patients.

**Methods:**

We conducted a cross-sectional study using 2017 Nationwide Ambulatory Surgery Sample.

Major ambulatory surgery encounters among patients aged ≥18 years with CD (n = 20,635) or UC (n = 9,894) were compared to 9.4 million encounters among non-IBD patients. Weighted percentages of patient characteristics (age, sex, median household income, primary payers, patient location, selected comorbidities, discharge destination, type of surgeries) and hospital-related characteristics (hospital size, ownership, location and teaching status, region) were compared by IBD status (CD, UC, and no IBD). Linear regression was used to estimate mean total charges, controlling for these characteristics.

**Results:**

Compared with non-IBD patients, IBD patients were more likely to have private insurance, reside in urban areas and higher income zip codes, and undergo surgeries in hospitals that were private not-for-profit, urban teaching, and in the Northeast. Gastrointestinal surgeries were more common among IBD patients. Some comorbidities associated with increased risk of surgical complications were more prevalent among IBD patients. Total charges were 9% lower for CD patients aged <65 years (Median: $16,462 vs $18,106) and 6% higher for UC patients aged ≥65 years (Median: $16,909 vs $15,218) compared to their non-IBD patient counterparts.

**Conclusions:**

Differences in characteristics of major ambulatory surgery encounters by IBD status may identify opportunities for efficient resource allocation and positive surgical outcomes among IBD patients.

## Introduction

Inflammatory bowel disease (IBD), including Crohn’s disease (CD) and ulcerative colitis (UC), involves chronic inflammation of the gastrointestinal (GI) tract. The prevalence of IBD has increased worldwide in the recent decades and will continue to rise [1]. In 2015, an estimated 3 million US adults reported having been told that they had either CD or UC [2]. IBD is a lifelong chronic condition with overall mortality rate similar to that among the US general population [3]. However, the disease’s systemic inflammation and side effects of medications often lead to the occurrence of extraintestinal comorbidities and complications among patients with IBD that affect nearly all organs, especially including musculoskeletal, dermatologic, and ocular systems [4]. A previous study based on the National Health Interview Survey (NHIS) showed that adults with IBD were more likely to have comorbidities than those without IBD [5].

A study based on multiple national surveys showed that IBD-related emergency department (ED) visits and hospital discharges increased significantly from 2005 through 2016, in spite that it might be attributable to the availability of more advanced medical treatments and biologic therapies in the recent decades [6]. Another NHIS study also found that overall health care use, including but not limited to hospitalizations, office visits, ED visits, surgeries, and medical procedures, was greater among patients with IBD compared with those without IBD [7]. Therefore, more health care resources are expected to be consumed by IBD patients for all diagnoses and treatments whether or not they are directly related to IBD care.

Characteristics in inpatient surgeries among IBD patients have been well studied [8–11]; however, to date, overall ambulatory surgeries performed on IBD patients have not been explored. An ambulatory surgery is a planned outpatient procedure for which a hospitalization is not required. Currently, over 5,000 Medicare-accredited ambulatory surgery centers are located in all states, District of Columbia (DC), and territories, and are concentrated in states with large populations [12]. Centers for Medicare and Medicaid Services use the Hospital Outpatient Prospective Payment System for reimbursement of surgeries performed from hospital outpatient departments based on Healthcare Common Procedure Coding System (HCPCS) codes [13,14]. Recently, there has been an increasing trend of shifting surgeries from inpatient to outpatient settings with feasibility of performing some surgeries in outpatient settings that were previously conducted in inpatient settings. The advantages of that include health care cost savings and convenience for patients [15,16], given comparable surgical outcomes [17]. Digestive system-related surgeries are commonly performed procedures in the outpatient settings, accounting for 14% of total ambulatory surgeries [18]. For overall disease management, it is important to understand the pattern of digestive disease- and non-digestive disease-related major ambulatory surgeries to help assess surgical needs for patients with IBD and timely ambulatory care may prevent more expensive acute care utilization. In addition, detailed ambulatory surgery data at the population level, including differences in characteristics associated with ambulatory surgery by IBD status, is important for health care resource planning in areas with high prevalence of IBD and identifying high-risk factors to reduce postsurgical complications to guide more efficient care for patients with IBD.

In this study, we sought to 1) determine whether patient- and hospital-related characteristics differed for any major ambulatory surgery performed on patients by IBD status (CD, UC, and no IBD), 2) describe type of major ambulatory surgeries by IBD status, and 3) compare mean total charges for major ambulatory surgery performed on patients with IBD versus those without IBD by using a national survey.

## Materials and methods

### Data source

We used the 2017 Healthcare Cost and Utilization Project (HCUP) Nationwide Ambulatory Surgery Sample (NASS), which is the largest all-payer ambulatory surgery database in the United States and sponsored by the Agency for Healthcare Research and Quality [19]. NASS is sampled from the State Ambulatory Surgery and Services Databases (SASD) containing community nonrehabilitation hospitals with general acute care service type, in which 32 states and the DC participated in 2017. These states are geographically dispersed and included 2,733 hospital-owned facilities in 2017, accounting for an estimated 61% of the entire hospital-owned facilities and 73% of the entire ambulatory surgery encounters in the US. The sampling strata were based on important characteristics (US regions, hospital bed size, urban-rural location of the hospital, hospital teaching status, and hospital ownership or control). The sampling weights were developed based on the NASS universal hospitals and encounters with the NASS sample hospitals and encounters to ensure that estimates are nationally representative. In 2017, the NASS produced weighted estimates of approximately 10.3 million major ambulatory surgery encounters in the US [20].

### Study population

We included encounters that occurred in 2017 among patients aged ≥18 years. Crohn’s disease (CD) and ulcerative colitis (UC) were identified if the *International Classification of Diseases*, *Tenth Revision*, *Clinical Modification* (ICD-10-CM) codes of K50 and K51, respectively, were present as the principal diagnosis or any secondary diagnosis. Of the encounters associated with IBD, 0.18% coded as both CD and UC were excluded from the analytic sample. Encounters with no listed IBD diagnosis codes were considered as non-IBD encounters.

### Major ambulatory surgery

Defined by NASS, major in-scope ambulatory surgeries (hereafter referred to as major ambulatory surgeries) are selected invasive, therapeutic surgeries that require the use of an operating room and general or regional anesthesia or sedation [20]. Diagnostic procedures such as endoscopies were not included. NASS uses HCUP Clinical Classification Software (CCS) procedure categories that are based on Current Procedural Terminology (CPT) or HCPCS level I codes. To define in-scope CCS procedure categories, the procedures are selected if they have relatively high major ambulatory surgery volumes, a substantial share of major surgeries performed in hospital-owned facilities and confirmed reliable reporting from hospitals in the SASD. In 2017, there were 78 in-scope CCS procedure categories [20]. We determined the most commonly performed procedures with encounters associated with CD or UC as the principal diagnosis or any-listed diagnosis or those not associated with IBD, stratified by age group.

### Variables

Patient demographic characteristics included age group (18–64 and ≥65 years), sex, quartile of median household income for patients’ zip code ($1 –<$43,000, $43,000 –<$54,000, $54,000 –<$71,000, and ≥$71,000), primary payer (Medicare, Medicaid, private insurance, self-pay, and others or no charge), patient residential location (urban and rural), and discharge destination (routine; transfer to short-term hospital or skilled nursing facility, intermediate care facility, or other facility; home health care; and others, including deceased or discharged against medical advice or unknown). Comorbidities were evaluated if they are risk factors for ambulatory surgery complications (depression, anxiety disorder, tobacco use disorder, opioid use disorder, alcohol use disorder, chronic obstructive pulmonary disease [COPD], obstructive sleep apnea, hypertension, and obesity) [21–24]. Comorbidities were defined according to the algorithm of chronic diseases from the Chronic Disease Warehouse [25], by searching any-listed diagnosis codes. Hospital-related characteristics included hospital size (number of beds [small: <100 beds, medium: from 100 to 300 beds, and large: ≥300 beds]), hospital ownership (government nonfederal, private not-for-profit, and private investor-owned), hospital location and teaching status (rural, urban nonteaching, and urban teaching), and hospital region (Northeast, Midwest, South, and West). We also included total charges ($) associated with encounters.

### Statistical analysis

We conducted analyses separately by IBD status (CD, UC, and no IBD). Descriptive statistics included percentages and 95% confidence intervals calculated for each patient- and hospital-related characteristic. Total charges for major ambulatory surgery encounters (mean with standard error and median with interquartile range) were calculated by IBD status, stratified by age group. Pairwise comparisons between IBD status categories were conducted by using t-tests by specifying linear contrasts. To account for 11% encounters with missing total charges in the 2017 NASS, we used a weighted sequential hot deck imputation approach to impute the missing values of total charges [26]. We first transformed total charges by natural logarithm to achieve normality because the original distribution was skewed, and then constructed a multivariable linear regression with a generalized estimating equation to estimate log-transformed total charges by IBD status, accounting for the correlation within hospital facilities and adjusting for aforementioned patient- and hospital-related characteristics. Significance level was determined at *P* < 0.05. The analyses were performed by using SAS-callable SUDAAN 11.0.3 (Research Triangle Institute, Research Triangle Park, North Carolina) to take weights and complex survey design into account to produce national estimates.

The NASS are limited data sets in which direct identifiers, as specified in the HIPAA Privacy Rule, have been removed. Therefore, Institutional Review Board approval was not required for this study.

## Results

In 2017, approximately 3 in 1,000 major ambulatory surgeries were performed on IBD patients aged ≥18 years in the United States (Table 1). Patients in CD encounters (CD patients) were more likely to be women and aged 18–64 years compared with patients in UC encounters (UC patients) or non-IBD encounters (non-IBD patients). UC patients had a higher proportion living in zip codes in the highest quartile of median household income (≥$71,000) compared with CD patients, who in turn had a higher proportion than non-IBD patients. In addition, both CD and UC patients were more likely to reside in urban areas than non-IBD patients. Both CD and UC patients also had a higher percentage of private insurance as their primary payer and a lower percentage of self-pay, charity, or no charge categories of primary payer than non-IBD patients. In addition, CD patients had a lower percentage of Medicare and UC patients had a lower percentage of Medicaid than non-IBD patients. UC patients were less likely to have routine discharge than CD patients who in turn were less likely to have routine discharge than non-IBD patients.

**Table 1:** Distributions of patient demographic and hospital characteristics associated with major in-scope^a^ ambulatory surgery encounters by inflammatory bowel disease status, 2017.

Characteristics	Crohn’s disease (CD)^b^ % (95% CI)	Ulcerative colitis (UC)^c^ % (95% CI)	No Crohn’s disease or ulcerative colitis % (95% CI)	*P* value (CD vs No IBD)	*P* value (UC vs no IBD)	*P* value(CD vs UC)
Unweighted encounter N	15,293	7,279	6,790,257	—	—	—
Weighted encounter N (%)	20,635 (0.2)	9,894 (0.1)	9,412,740 (99.7)	—	—	—
**Patient characteristics**						
**Age group 18–64 (years)**	75.0 (74.0–75.9)	64.6 (63.2–66.0)	64.9 (64.4–65.3)	<0.001	0.69	<0.001
**Women**	60.1 (59.2–61.1)	57.3 (56.0–58.6)	58.1 (57.8–58.3)	<0.001	0.24	<0.001
**Quartiles of median household income ($) at patient’s zip code level**						
1–<43,000	21.2 (19.9–22.6)	17.3 (15.9–18.7)	24.5 (23.6–25.4)	<0.001	<0.001	<0.001
43,000–<54,000	25.7 (24.5–26.9)	24.0 (22.3–25.8)	27.5 (26.7–28.3)	<0.001	<0.001	0.03
54,000–<71,000	26.7 (25.6–27.8)	26.9 (25.6–28.3)	25.5 (24.9–26.2)	0.01	0.02	0.07
≥71,000	26.4 (24.6–28.3)	31.8 (29.4–34.3)	22.5 (21.3–23.7)	<0.001	<0.001	<0.001
**Urban patient residential location**	83.0 (81.8–84.3)	84.4 (82.6–86.0)	79.4 (78.5–80.2)	<0.001	<0.001	0.07
**Primary payer**						
Medicare	31.0 (30.0–32.1)	34.9 (33.5–36.3)	35.7 (35.2–36.2)	<0.001	0.23	<0.001
Medicaid	10.9 (10.2–11.7)	6.5 (5.8–7.3)	11.1 (10.7–11.6)	0.56	<0.001	<0.001
Private insurance	53.6 (52.4–54.8)	54.3 (52.7–55.8)	46.2 (45.5–46.8)	<0.001	<0.001	0.41
Self-pay	1.5 (1.2–1.7)	1.5 (1.2–1.9)	2.6 (2.3–2.8)	<0.001	<0.001	0.72
Others or no charge	3.0 (2.7–3.4)	2.9 (2.4–3.3)	4.5 (4.3–4.7)	<0.001	<0.001	0.50
**Discharge destination**						
Routine	89.0 (86.0–91.1)	85.5 (81.7–88.7)	91.4 (90.1–92.5)	0.001	<0.001	<0.001
Transfer	0.4 (0.3–0.5)	0.4 (0.3–0.6)	0.4 (0.4–0.5)	0.33	0.81	0.74
Home health care	0.5 (0.4–0.7)	0.6 (0.4–0.8)	0.5 (0.5–0.6)	0.79	0.31	0.49
Other^d^ or unknown	10.1 (8.0–12.5)	13.5 (10.3–17.4)	7.7 (6.5–8.9)	0.001	<0.001	0.001
**Comorbidities**						
Chronic obstructive pulmonary disease	6.5 (6.0–7.0)	5.7 (5.1–6.3)	4.8 (4.7–5.0)	<0.001	0.01	0.02
Obstructive sleep apnea	5.2 (4.7–5.6)	6.9 (6.3–7.6)	4.2 (4.0–4.4)	<0.001	<0.001	<0.001
Hypertension	35.8 (34.7–36.9)	39.8 (38.4–41.2)	37.4 (36.8–38.1)	0.003	0.002	<0.001
Obesity	11.8 (11.1–12.6)	13.2 (12.3–14.2)	11.7 (11.3–12.2)	0.78	0.001	0.005
Depression	22.7 (21.7–23.7)	21.3 (20.1–22.5)	11.2 (10.8–11.7)	<0.001	<0.001	0.03
Anxiety disorder	15.4 (14.6–16.2)	14.6 (13.6–15.6)	7.3 (7.0–7.6)	<0.001	<0.001	0.14
Tobacco use disorder	14.8 (14.1–15.5)	7.9 (7.2–8.7)	10.6 (10.3–10.9)	<0.001	<0.001	<0.001
Opioids use disorder	0.3 (0.2–0.4)	—^e^	0.1 (0.1–0.1)	<0.001	—	—
Alcohol use disorder	0.4 (0.3–0.5)	0.5 (0.3–0.7)	0.3 (0.3–0.3)	0.22	0.12	0.51
**Hospital characteristics**						
**Hospital size**						
Small (<100 beds)	11.4 (10.0–12.9)	11.2 (9.4–13.3)	15.5 (14.5–16.5)	<0.001	<0.001	0.78
Medium (100–<300 beds)	33.1 (30.4–35.9)	37.0 (33.4–40.8)	35.3 (33.5–37.2)	0.02	0.27	0.001
Large (≥300 beds)	55.5 (52.5–58.5)	51.8 (47.9–55.7)	49.2 (47.3–51.1)	<0.001	0.10	0.003
**Hospital ownership**						
Government, nonfederal	9.5 (7.7–11.7)	7.8 (6.2–9.8)	12.1 (10.7–13.6)	<0.001	<0.001	0.01
Private, not-for-profit	83.4 (81.0–85.5)	84.8 (82.4–87.0)	76.5 (74.7–78.1)	<0.001	<0.001	0.08
Private, investor owned	7.1 (6.0–8.3)	7.4 (6.0–8.9)	11.4 (10.5–12.4)	<0.001	<0.001	0.55
**Hospital location and teaching status**						
Rural	9.4 (8.2–10.7)	9.3 (7.6–11.2)	13.8 (13.0–14.6)	<0.001	<0.001	0.86
Urban non-teaching	20.1 (18.3–22.0)	20.5 (18.1–23.1)	24.1 (23.0–25.2)	<0.001	0.001	0.67
Urban teaching	70.5 (68.2–72.7)	70.2 (67.1–73.2)	62.1 (60.8–63.5)	<0.001	<0.001	0.79
**Hospital region**						
Northeast	20.6 (18.3–23.1)	22.2 (19.0–25.9)	17.6 (16.3–18.9)	0.001	0.003	0.14
Midwest	27.8 (25.1–30.6)	26.2 (23.1–29.6)	26.3 (25.0–27.5)	0.16	0.98	0.09
South	36.3 (33.6–39.1)	30.4 (27.5–33.6)	36.3 (34.8–37.8)	0.97	<0.001	<0.001
West	15.3 (13.4–17.4)	21.1 (18.0–24.6)	19.9 (18.6–21.3)	<0.001	0.38	<0.001

CI, confidence interval; CD, Crohn’s disease; UC, ulcerative colitis; IBD, inflammatory bowel disease.

^a^ In-scope major surgeries refer to relatively high major ambulatory surgery volumes, a substantial share of major surgeries performed in hospital-owned facilities, and confirmed reliable from hospitals in the State Ambulatory Surgery and Services Databases.

^b^ Any-listed ICD-10-CM diagnosis code of K50.

^c^ Any-listed ICD-10-CM diagnosis code of K51.

^d^ Other discharge destinations include dead or against medical advice. According to Data User Agreement of Healthcare Cost and Utilization Project (https://www.hcup-us.ahrq.gov/db/nation/nass/NASS_Introduction_2017.jsp), “other” was grouped with “unknown” due to the small sample size.

^e^ Data suppressed due to small sample size according to the HCUP data reporting rule.

A higher proportion of CD patients had COPD than did UC patients, who had a higher proportion with COPD than did non-IBD patients. A higher proportion of UC patients had obstructive sleep apnea than did CD patients, who had a higher proportion with obstructive sleep apnea than did non-IBD patients. The proportion of patients with hypertension was highest among UC patients and lowest among CD patients. The proportion with obesity was higher among UC patients than their counterparts. The proportion with depression and anxiety was higher among both CD and UC patients than non-IBD patients. Tobacco use disorder was least common among UC patients and most common among CD patients. Opioid use disorder was more common among CD patients than non-IBD patients. There was no significant association between IBD status and alcohol use disorder.

For hospital-related characteristics, major ambulatory surgeries on both CD and UC patients were more likely to take place in private not-for-profit hospitals, urban teaching hospitals, and hospitals in the Northeast, and less likely to take place in small hospitals, compared with surgeries on non-IBD patients. Finally, major ambulatory surgeries were less likely to occur in the West for CD patients and in the South for UC patients than their counterparts.

Most major ambulatory surgeries were performed among patients aged 18–64 years—75% among CD patients and 65% among UC and non-IBD patients (Table 1). When CD or UC was the principal diagnosis for both age groups, a vast majority of the major ambulatory surgeries were lower GI-related procedures (mainly incision procedures on anus or laparoscopic incision procedures on the intestines excluding anus) and colorectal resection (mainly excision of rectal tumours typically small and benign) (Table 2). When CD or UC was any-listed diagnosis, the most commonly performed procedures among patients aged 18–64 years included lower GI-related procedures and cholecystectomy and common duct exploration; among patients aged ≥65 years, lens and cataract procedures were most common.

**Table 2:** Types of major in-scope^a^ ambulatory surgeries with the top high-procedure volume by order, by age groups and inflammatory bowel disease status, 2017.

IBD status for the encounter	Age 18–64 weighted N^b^ (weighted %)	Age ≥65 weighted N^b^ (weighted %)
CD as the principal diagnosis	N = 1,311 Other operating room lower gastroenterology therapeutic procedures (87.2) Colorectal resection (2.1) Other operating room procedures on vessels other than head and neck (1.8)	N = 59 Other operating room lower gastroenterology therapeutic procedures (65.5)
UC as the principal diagnosis	N = 142 Other operating room lower gastroenterology therapeutic procedures (48.7) Other operating room procedures on vessels other than head and neck (15.9) Colorectal resection (10.3)	N = 51 Colorectal resection (32.0) Other operating room procedures on vessels other than head and neck (25.4) Other operating room lower gastroenterology therapeutic procedures^c^
CD as any-listed diagnosis	N = 15,478 Other operating room lower gastroenterology therapeutic procedures (20.7) Cholecystectomy and common duct exploration (7.5) Other hernia repair (5.2)	N = 4,953 Lens and cataract procedures (22.3) Other therapeutic procedures on muscles and tendons (5.1) Other hernia repair (4.7)
UC as any-listed diagnosis	N = 6,390 Cholecystectomy and common duct exploration (8.1) Other therapeutic procedures on muscles and tendons (6.7) Other operating room lower gastrointestinal therapeutic procedures (6.2)	N = 3,504 Lens and cataract procedures (22.5) Inguinal and femoral hernia repair (6.2) Other therapeutic procedures on muscles and tendons (5.3)
No IBD	N = 6,105,200 Other therapeutic procedures on muscles and tendons (7.5) Cholecystectomy and common duct exploration (7.4) Hysterectomy, abdominal and vaginal (4.8)	N = 3,307,540 Lens and cataract procedures (28.6) Insertion, revision, replacement, removal of cardiac pacemaker or cardioverter or defibrillator (6.3) Other therapeutic procedures on muscles and tendons (5.2)

IBD, inflammatory bowel disease; CD, Crohn’s disease; UC, ulcerative colitis.

^a^ Refers to relatively high major ambulatory surgery volumes, a substantial share of major surgeries performed in hospital-owned facilities and confirmed reliable from hospitals in the State Ambulatory Surgery and Services Databases. According to first-listed procedure.

^b^ Number of encounters weighted to the national level.

^c^ Percent suppressed due to small sample size according to the HCUP data reporting rule.

When adjusting for patient- and hospital-related characteristics, total charges for encounters were 9% lower among patients with CD aged 18–64 years and 6% higher among patients with UC aged ≥65 years compared with non-IBD patients in the corresponding age groups (Table 3).

**Table 3:** Total charges^a^ for major in-scope^b^ ambulatory surgery encounters by age and status of inflammatory bowel disease, 2017.

Total charges	No IBD	Crohn’s disease	*P* value^c^	Ulcerative colitis	*P* value^c^
**Age 18–64 years**					
Mean $ (SE)	25,224 (316)	22,937 (408)	--	24,704 (521)	--
Median $ (IQR)	18,106 (10,705–30,664)	16,462 (10,070–27,577)	--	18,363 (11,425–30,064)	--
Unadjusted^d^	Ref^e^	0.91 (0.89–0.93)	<0.001	1.01 (0.98–1.03)	0.65
Adjusted^f^	Ref^e^	0.91 (0.90–0.93)	<0.001	1.00 (0.98–1.03)	0.72
**Age ≥65 years**					
Mean $ (SE)	25,648 (374)	25,325 (668)	--	27,077 (869)	--
Median $ (IQR)	15,218 (8,739–30,126)	15,801 (8,739–30,126)	--	16,909 (9,135–31,462)	--
Unadjusted^d^	Ref^e^	1.01 (0.98–1.04)	0.56	1.07 (1.03–1.10)	<0.001
Adjusted^f^	Ref^e^	1.01 (0.98–1.03)	0.69	1.06 (1.03–1.10)	<0.001

IBD, inflammatory bowel disease; SE, standard error; IQR, interquartile range; Ref, referent group.

^a^ Approximately 11% missing total charges were imputed based on the weighted sequential hot deck method with IBD status, age group, sex, quartiles of median household income for patient’s zip code, urban patient residential location, primary payer, and discharge destination as donors.

^b^ Refers to relatively high major ambulatory surgery volumes, a substantial share of major surgeries performed in hospital-owned facilities and confirmed reliable.

^c^ Wald χ^2^ test for the association between IBD status and total charges (natural logarithm transformed).

^d^ Linear regression predicting total charges (natural logarithm) by IBD status. The estimates were back transformed in ratios.

^e^ The referent group is the encounters not associated with either Crohn’s disease or ulcerative colitis.

^f^ Adjusted for age, sex, all select comorbidities, median household income for patient’s zip code, patient residential location, primary payer, discharge destination, hospital size, hospital ownership, hospital location and teaching status, and hospital region.

## Discussion

Differences in patient and hospital characteristics of major ambulatory surgeries by IBD status were found. These findings may help identify opportunities for efficient resource allocation and warrant perioperative management for IBD patients.

The current study highlighted some important findings. First, GI-related ambulatory surgeries were more common among IBD patients, especially CD patients younger than 65 years, which reflects greater GI-related surgical needs among this age group because incidence of IBD usually peaks in one’s 20s and 30s [27]. Perianal disease, usually treated by an outpatient surgery, is known to be more commonly associated with CD than UC [28]. By examining the CPT codes associated with lower GI therapeutic procedures, the vast majority of procedures involved incision on anus and were most commonly performed on CD patients aged 18–64 years in the current study. When IBD was not the primary diagnosis, the major ambulatory surgeries performed among patients aged 65 years or older with IBD were similar to those without IBD.

Second, patients with IBD who had major ambulatory surgeries were more likely to reside in zip codes with a higher median household income than those without IBD. This finding is consistent with the results from a previous study by Park and colleagues using Medical Expenditure Panel Survey data that showed patients with IBD were more likely to have a higher family income [29]. In the current study, patients with IBD were more likely to have private insurance than patients without IBD, and the lower likelihood of having Medicaid among UC patients may be associated with higher household income. However, patients with IBD who were uninsured or underinsured may be less likely to have access to ambulatory surgery service. Although we were not able to assess the association between income level and likelihood of receipt of ambulatory surgeries among patients with IBD in the current study, Park and colleagues further showed that patients with IBD living in poverty were less likely to have outpatient care than those not living in poverty, which could cause higher likelihood of subsequent hospitalization or ED visits [29].

Third, patients with IBD who underwent major ambulatory surgeries were more likely to reside in urban areas than those without IBD. However, the IBD prevalence is similar in rural areas compared with urban areas [2]. It is possible that patients with IBD who live in rural areas have limited access to outpatient surgeries than rural patients without IBD. Our previous national study demonstrated that IBD-related office visit rates were lower in rural areas than urban areas whereas CD-specific hospitalization and ED visit rates were higher in rural than urban areas [30]. Lower outpatient access was found to increase the likelihood of hospitalization or ED visit, and subsequently increase the healthcare costs [29]. The findings suggest that it may be important to have efficient resource allocations, including prioritizing ambulatory surgery centers and medical supplies, in rural areas with a high density of IBD population.

Fourth, patients with IBD, especially UC, were less likely to have routine discharge compared with patients without IBD. Because we were not able to assess individual discharge status because of the small sample sizes, it is unknown whether destination of transfer, home health care, discharge against medical advice, or death were related to surgical complications. Furthermore, the results showed that IBD patients were more likely to have comorbidities, which might also have influenced their discharge status. Comorbidities, such as obesity, hypertension, COPD, history of stroke or transit ischemic attack, and obstructive sleep apnea, have been observed to be associated with increased risk of morbidities and mortalities following outpatient day surgery [23,24]. Except for history of stroke or transient ischemic attack that were not included in these analyses because of small sample size, our results showed a higher prevalence of almost all the conditions assessed among patients with IBD compared with patients without IBD. Patients with CD and UC had the highest prevalence of COPD and obstructive sleep apnea, respectively. In addition to these conditions, depression and anxiety are also prevalent among IBD patients [22], and the prevalence among patients with IBD was twice that among patients without IBD in this study. Furthermore, mental illness is also correlated with substance use disorders, which are also prevalent among persons with IBD; one in six persons with IBD has a substance use disorder [21]. Narcotic drugs have been used to control pain in patients with IBD who may be susceptible to overdose later. Substance use disorders and poor mental health have been shown to be associated with adverse surgical and anesthetic events and complications [31–33]. As these comorbidities are more common among IBD patients, it may be important for surgeons and anesthesiologists to have pre- and post-surgical evaluations, such as risk assessment, counseling, and referral for follow-up, to prevent complications and improve surgical outcomes. In addition, future research that explores the quality of ambulatory surgeries (e.g., perioperative assessment and patient follow-up) and evaluation of outcomes (e.g., surgical complications and discharge destination) by IBD status may be warranted to further examine potential differences in surgical outcomes.

The current study also noted that the total charges of major ambulatory surgeries differed by IBD status and age group. The cause of the observed differences is likely multifactorial. The unadjusted and adjusted models yielded similar results, indicating that total charges might not be primarily driven by patient or hospital characteristics, rather by bills of relevant procedures charged by hospitals. CD patients tended to be younger in the study and their primary major ambulatory encounters were more likely to be a lower gastroenterology therapeutic procedure compared with their counterparts. An ad-hoc analysis showed that the average total charges for three most frequently performed procedures was lower among CD patients than non-IBD patients which may indicate that type of procedures could influence total charges. Among older IBD patients, gastroenterology-related surgeries were not the most commonly performed procedures. Instead, age-related procedures, such as lens or cataract and muscles or tendons, were the most common procedures, regardless of IBD status. Notably, although total charges did not differ between CD and non-IBD patients in the older group, they were higher among UC patients than their non-IBD counterparts. It was possible that certain major ambulatory surgeries in higher demand among older UC patients were associated with a higher charge than those for their non-IBD counterparts. Besides type of surgeries, other factors, such as provider type, that were not able to be examined in this study might also influence total charges. Furthermore, the NASS does not have cost-to-charge ratio adjustment as costs could provide a more consistent comparison across hospitals. Nonetheless, total charges provide information about economic impact on major ambulatory surgery encounters by IBD status. Future studies to explore costs with follow-up visits are also warranted as IBD patients are more susceptible than non-IBD patients to postsurgical complications that may incur higher health care costs subsequently. Other than total charges difference, there were some differences in hospital-related characteristics. For instance, consistent with previous study findings, patients with IBD were more likely to undergo care in hospitals in the Northeast region, and patients with UC were more likely to be cared for in hospitals in the West region [34,35]. In addition, patients with IBD were more likely to have major ambulatory surgeries in private not-for-profit hospitals. A previous study indicated that hospital ownership played a role in health care-related outcomes in terms of lower payment and mortality in private not-for-profit hospitals [36].

To our knowledge, this was the first nationwide study describing characteristics of major ambulatory surgeries among IBD patients in the United States. The biggest strength is the survey’s large sample size to study all-payer major ambulatory surgeries among patients with IBD at the national level. There are several limitations in the study. First, the NASS data are at the encounter level, not the patient level. Multiple encounters with the same patient cannot be identified. Second, the data do not contain HCPCS level II codes in the 2017 NASS. Therefore, data on specific procedures could not be obtained. Third, the lack of a patient race or ethnicity variable in the 2017 NASS prevented us from assessing any differences in major ambulatory surgery by race or ethnicity. Fourth, although the NASS produces national estimates based on hospital-owned facilities, non-hospital affiliated ambulatory surgery centers are not included in the NASS. Finally, cost-to-charge ratio data were unavailable; therefore, total charges could be overestimated compared with the actual costs.

## Conclusions

The current study indicated a higher need of GI-related major ambulatory surgeries among patients with IBD, a higher prevalence of certain comorbidities associated with IBD patients, and a higher prevalence of IBD-related ambulatory surgeries in certain geographic regions compared with non-IBD patients from this ambulatory surgery population. These differences by IBD status can inform healthcare professionals and policy makers when considering service planning and clinical decision-making during perioperative management for IBD patients. Future research that examines the socioeconomic characteristics associated with likelihood of receipt of ambulatory surgeries among the IBD population may provide more insight into the income differences we observed. Further exploration of ambulatory surgery resource allocation in certain geographic areas might be important to improve access to outpatient care especially among IBD patients with lower socioeconomic status.
